# Comparative Effectiveness of Direct Oral Anticoagulants and Warfarin on Venous Thromboembolism in Cancer Patients

**DOI:** 10.1002/cam4.71209

**Published:** 2025-09-09

**Authors:** Hye‐In Jung, Claudia Geue, Giorgio Ciminata, Eui‐Kyung Lee

**Affiliations:** ^1^ School of Pharmacy Sungkyunkwan University Suwon South Korea; ^2^ Health Economics and Health Technology Assessment (HEHTA), University of Glasgow Glasgow UK

**Keywords:** anticoagulants, neoplasms, venous thromboembolism

## Abstract

**Introduction:**

Venous thromboembolism (VTE) is a leading cause of mortality in cancer patients, and a substantial number of patients are being treated with oral anticoagulants. We aim to assess the comparative effectiveness of direct oral anticoagulants (DOACs) compared to warfarin for VTE treatment in cancer patients.

**Methods:**

In this retrospective cohort study, we included 2,367 cancer patients who are new users of oral anticoagulants (OACs) for VTE treatment from 2009 to 2021 in NHS Scotland. Patients were grouped by OAC type, DOACs or warfarin. To adjust for confounding, inverse probability treatment weighting was applied. Outcomes included mortality, VTE recurrence, and major bleeding. We calculated Hazard Ratio (HR) using Cox regression and sub‐distribution HR (sHR) using a competing risk framework (Fine and Gray method) for VTE recurrence and major bleeding. Subgroup analyses were conducted for individual DOACs.

**Results:**

Patients on DOACs had lower risks of VTE recurrence (sHR 0.73 95% CI 0.59–0.90) and major bleeding (sHR 0.68, 95% CI 0.53–0.88) compared to patients on warfarin. Patients on rivaroxaban (HR 1.21, 95% CI 1.04–1.39) and edoxaban (HR 1.59, 95% CI 1.15–2.22) had a significantly higher risk of mortality, while a comparable risk of mortality was observed (HR 0.91, 95% CI 0.76–1.08) for patients on apixaban compared to patients on warfarin.

**Conclusion:**

This study provides insight into the effectiveness of DOACs compared to warfarin for VTE in cancer patients. Patients on DOACs had lower risks of VTE recurrence and major bleeding. We suggest healthcare professionals consider the potential benefits of individual DOACs when making treatment decisions.

## 1 Introduction

Venous thromboembolism (VTE) is a life‐threatening complication that is recorded to be the cause of death in 3.5% of cancer patients [1, 2]. VTE is strongly associated with cancer, and about 18% of newly diagnosed VTE was reported to be attributed to underlying cancer [3]. Other literature also corroborated the association and showed that 8.4% of patients who were hospitalized with cancer were reported to have VTE [4].

The risk of developing VTE during cancer treatment may depend on patient‐related, tumor‐related, or treatment‐related factors [5]. Due to increased blood coagulability resulting from cancer cell release, cancer patients are at an increased risk of VTE [6]. Cancers associated with a higher incidence of VTE include brain, pancreas, stomach, bladder, gynecological, lung, lymphoma, myeloproliferative neoplasm (MPN), kidney, and testicular cancer. However, there are some cancer types where a lower incidence of VTE can be observed compared to patients without cancer, such as prostate and breast cancer [5, 7]. Prevalent anticancer therapy such as chemotherapy, immunotherapy, antiangiogenic therapy, protein kinase inhibitors, and supportive care drugs are also risk factors for VTE [8]. In addition, an established VTE history is a risk factor for recurrent VTE and bleeding in cancer patients, despite anticoagulation [9].

While low molecular weight heparin (LMWH) has long been recommended over oral anticoagulants (OACs) for VTE in cancer patients [5], emerging evidence has demonstrated that direct oral anticoagulants (DOACs) offer non‐inferior results in terms of VTE recurrence and major bleeding compared to dalteparin in cancer patients [10, 11, 12]. Consequently, clinical guidelines have been updated to recommend DOACs at the same level as LMWH for treating cancer‐associated VTE. However, these guidelines acknowledged that their recommendation is based on low to very low‐quality evidence, primarily due to the lack of inclusion of high‐risk patients [13, 14, 15, 16].

A substantial proportion (48.1%–81%) of patients with cancer‐associated thrombosis are treated with OACs for convenience in administration and to minimize the risk of discontinuing the treatment [17, 18, 19]. While OACs have been increasingly utilized for VTE treatment in cancer patients, questions remain around the comparative effectiveness of warfarin vs. DOACs [20]. In a recent study, warfarin was suggested to be associated with better overall survival (OS) compared to DOACs in elderly (over 65 years) cancer patients with VTE [21]. This study included only elderly patients with cancers associated with a high risk of VTE. In contrast, several studies have demonstrated that DOACs are associated with a lower risk of VTE recurrence and major bleeding [22, 23]. These studies tend to focus on apixaban only or have short follow‐up periods, making survival assessment challenging.

Our study therefore aims to address this gap in evidence by assessing the effectiveness of DOACs compared to warfarin for VTE treatment in cancer patients in NHS Scotland. We will be able to consider factors that have not been considered in previous studies, including patient‐related, tumor‐related, and treatment‐related factors to evaluate outcomes for cancer patients in real‐life clinical practice using routinely collected data.

## 2 Materials and Methods

### 2.1 Data Source and Study Setting

This study is a retrospective cohort study using administrative linked health datasets from NHS Scotland. The cohort population consists of cancer patients who are new users of DOACs or warfarin for their VTE treatment. This study involved data from Jan 2008 to May 2021. All patients who were treated with warfarin or DOACs from 12 Dec 2011 to Nov 2020, the index period, were identified by the Scottish Prescribing Information System (PIS). The start of the index period aligns with the approval of the first DOAC (apixaban) for VTE treatment in NHS Scotland [24]. The index date was set to be the first date of either a DOAC or warfarin prescription. To examine health status and medication use prior to the index date, individuals' records were included from 1 year prior to the index date as the pre‐index period. In order to allow for a minimum follow‐up of 6 months, the index period ended in Nov 2020. The follow‐up period started at the index date until either the date of death or the end of the study.

The PIS database includes all prescribed medicines from community pharmacies, practice prescribers, health boards, and specialist appliance suppliers [25]. General Acute Inpatient and Day Case Scottish Morbidity Records 01 (SMR01), mortality records, and demographic data were linked to PIS at an individual level. SMR01 includes all inpatient stays and day cases that are discharged from hospitals except for obstetric and psychiatric specialties [26]. From SMR01, we identified clinical events using the International Classification of Diseases, 10th Revision, Clinical Modification (ICD‐10), and OPCS‐4 codes (Table S1) [27].

### 2.2 Study Population

**FIGURE 1:**
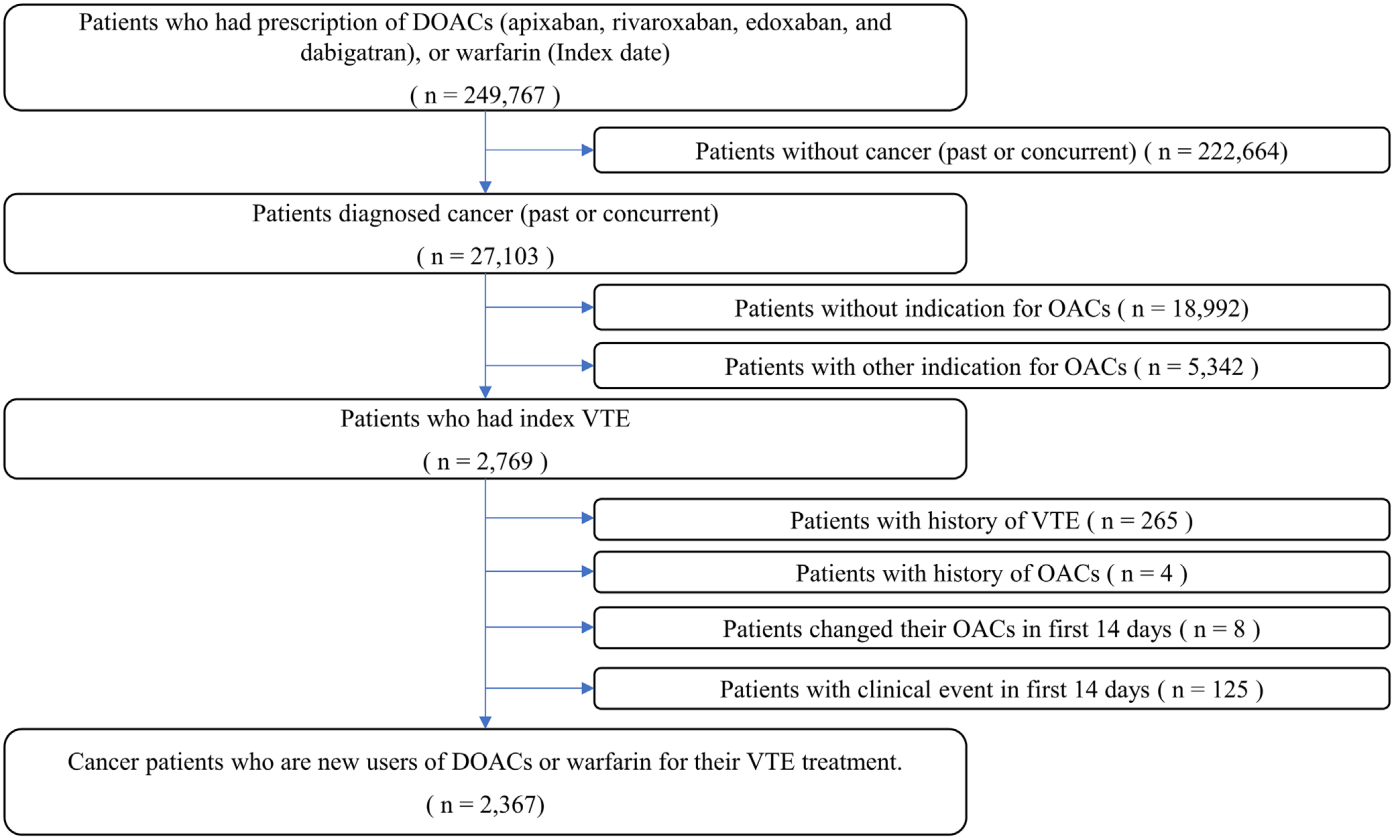
Study scheme. DOACs, direct oral anticoagulants; OAC, oral anticoagulant; VTE, venous thromboembolism.

This study includes cancer patients who are new users of DOACs or warfarin for their VTE treatment (Figure 1). The specific inclusion criteria are:

1. Patients with a 1st prescription of DOACs (apixaban, rivaroxaban, edoxaban, or dabigatran) or warfarin.
2. Patients who had a diagnosis of cancer any time before the index date or within 90 days after the index date.
3. Patients who had their 1st symptomatic or asymptomatic VTE (index VTE) within 30 days before the index date.

The study cohort excluded:

1. Patients with other indications for OACs than VTE (i.e., Atrial Fibrillation, Mitral stenosis, valvular disease, and heart valve replacement) within 30 days before the index date.
2. Patients with a history of VTE during the pre‐index period.
3. Patients who had any type of OACs during the pre‐index period.
4. Patients who had any clinical events within 14 days after the index date.

New users were defined as patients with a 1st prescription of a DOAC or warfarin during the index period without any use of the drug during the pre‐index period [28]. An intention‐to‐treat (ITT) approach was applied. However, to ascertain the effect of OACs, patients who changed the type of OAC or experienced any of the study outcomes during the first 14 days of the follow‐up period (grace period) were excluded. This grace period allows for long‐acting anticoagulants to conduct bridging therapy with shorter‐acting anticoagulants, which are often required and could affect clinical outcomes in the short term [29].

### 2.3 Outcomes

The effectiveness of DOACs compared to warfarin was compared using the following outcomes: mortality, VTE recurrence, and major bleeding events. VTE recurrence is strongly associated with a poor prognosis in terms of mortality and morbidity [30]. Major bleeding is one of the most serious adverse drug reactions to anticoagulants [31]. It includes intracerebral hemorrhage, subarachnoid hemorrhage, subdural hemorrhage, and upper and lower gastrointestinal bleeding, all requiring hospitalization [27, 32]. The time to respective events is set to be the number of days from the index date to the date of the clinical event.

### 2.4 Covariates

Patient‐related factors included age, sex, socioeconomic status, type of VTE, the Charlson comorbidity index (CCI), and risk scores. Age was measured in 5‐year bands with consolidation of two ends, under 60 and over 85. The Scottish Index of Multiple Deprivation (SIMD) was categorized into quintiles (quintile 1 = most deprived areas; quintile 5 = least deprived areas) and captures socioeconomic status. VTE type was classified as (i) deep vein thrombosis (DVT) or (ii) pulmonary embolism (PE). The Charlson comorbidity index (CCI) was applied with the cancer weight excluded and categorized into no, one or two, and three or more comorbidities [33]. Two risk scores, COMPASS‐CAT and HAS‐BLED, were included. COMPASS‐CAT evaluates the risk of cancer‐associated thrombosis (CAT) in patients with cancer by using cancer treatment and advanced status, predisposing risk factors for VTE, and platelet count [34]. HAS‐BLED assesses bleeding risk in patients with anticoagulation [35]. To generate the COMPASS‐CAT risk score, the advanced stage of cancer variable was counted if the patients had a diagnosis of secondary malignant neoplasm or multiple primary malignant neoplasms, and high platelet count as to thrombocythemia or thrombocytosis. Disease codes to identify components of the comorbidity and risk scores were referenced from relevant literature [36, 37, 38]. Tumor‐related factors included the classification of cancer type based on VTE risk (Table S2). Treatment‐related factors included cancer activity. Cancer was assumed to be active when there was a record of cancer diagnosis or treatment‐related to cancer between 90 days before and after the index date [39].

### 2.5 Statistical Methods

To account for confounding in this observational study, the imbalance of baseline characteristics was examined using propensity score (PS) estimation. Propensity scores were estimated using a logit model, and the included covariates were age group, sex, SIMD quintile, cancer activity, type of cancer, CCI, HAS‐BLED, and COMPASS‐CAT. The distribution of propensity scores showed a notable imbalance between the two groups. Assuming the absence of unobserved confounding, different adjusting methods were tested, including PS matching, nearest neighbor (NN) matching with exact matching on cancer type, and inverse probability treatment weighting (IPTW), to balance the covariates and obtain the average treatment effect (ATE) (Figure S1). As the proportion of missing data was < 5%, missing values were not imputed [40]. Standardized differences were used to assess balance [41]. IPTW showed negligible standardized differences, below 0.1 for all covariates, and did not lead to a loss of observations [42]. We used IPTW to adjust for observed confounding, which allows for an unbiased estimation of ATE [43].

Kaplan–Meier estimates were generated for all outcomes. Hazard ratios were estimated using Cox proportional hazard models, adjusting for all covariates included in PS estimation, using the double‐robust method [44]. Where the proportional hazard assumption was violated, a time‐varying effect was included. All outcomes were regarded to be competing with death, and cumulative incidence functions and sub‐distribution hazard ratios were obtained using the Fine and Gray method [45].

### 2.6 Subgroup Analysis

Subgroup analyses were conducted for the different types of DOACs. For potential differences in effectiveness among DOACs, we separately compared the effectiveness of rivaroxaban, apixaban, and edoxaban to the effectiveness of warfarin. The same weights were applied in these analyses.

All analyses were performed using STATA version 16.0 software (STATA Corporation, College Station, TX, USA).

### 2.7 Ethics

This study did not require any consenting/contacting patients directly; no ethics approval was sought. This study used pseudonymized data; data reported are aggregated to minimize the risk of identification, and output clearance is required.

## 3 Results

2367 cancer patients were included who were new users of DOAC or warfarin for their VTE treatment (Figure 1). The median follow‐up was 23.2 months. In the warfarin group, 48.09% were female with a mean age of 70.48 years. In the DOACs group, 54.61% were female with a mean age of 71.23 years. In both groups, the type of VTE was mainly PE. Among patients on DOACs, rivaroxaban (50.70%) was the most frequently prescribed drug, followed by apixaban (42.11%), edoxaban (6.89%), and dabigatran (0.29%). About half of the patients had active cancer (49.85%) (Table 1).

**TABLE 1:** Baseline characteristics of cancer patients with oral anticoagulants for VTE.

Covariates	DOACs		Warfarin		Total	
	(*N* = 1712)		(*N* = 655)		(*N* = 2367)	
	*N*	%	*N*	%	*N*	%
**Sex**						
Male	777	45.39	340	51.91	1117	47.19
Female	935	54.61	315	48.09	1250	52.81
**Age, mean (SD)**	71.23	12.13	70.48	11.63	71	11.99
Under 60	262	15.30	111	16.95	373	15.76
60–64	172	10.05	77	11.76	249	10.52
65–69	247	14.43	93	14.20	340	14.36
70–74	306	17.87	104	15.88	410	17.32
75–79	288	16.82	120	18.32	408	17.24
80–84	213	12.44	95	14.50	308	13.01
Over 85	224	13.08	55	8.40	279	11.79
**Type of VTE**						
DVT	442	25.82	215	32.82	657	27.76
PE	1270	74.18	440	67.18	1710	72.24
**Cancer activity**						
Yes	821	47.96	359	54.81	1180	49.85
No	891	52.04	296	45.19	1187	50.15
**Cancer type**						
High risk	591	34.52	210	32.06	801	33.84
Low risk	485	28.33	187	28.55	672	28.39
Others	636	37.15	258	39.39	894	37.77
**SIMD**						
1 (Most deprived)	313	18.28	115	17.56	428	18.08
2	357	20.85	155	23.66	512	21.63
3	369	21.55	139	21.22	508	21.46
4	330	19.28	129	19.69	459	19.39
5 (Least deprived)	338	19.74	115	17.56	453	19.14
Not answered	5	0.29	< 5	< 0.76	—	—
**CCI, mean (SD)**	0.81	1.34	1.07	1.64	1	1.44
0	1040	60.75	351	53.59	1391	58.77
1–2	485	28.33	203	30.99	688	29.07
3+	187	10.92	101	15.42	288	12.17
**HAS‐BLED, mean (SD)**	2.73	1.53	2.98	1.58	3	1.55
0–2 (low to moderate risk)	772	45.09	267	40.76	1039	43.90
3+ (moderate to high risk)	940	54.91	388	59.24	1328	56.10
**COMPASS‐CAT, mean (SD)**	7.27	4.53	7.96	4.57	7	4.55
0–6 (low to moderate risk)	689	40.25	241	36.79	930	39.29
7+ (high risk)	1023	59.75	414	63.21	1437	60.71
**Type of index OAC**						
Warfarin	—	—	655	100.00	655	27.67
Rivaroxaban	868	50.70	—	—	868	36.67
Apixaban	721	42.11	—	—	721	30.46
Edoxaban	118	6.89	—	—	118	4.99
Dabigatran	5	0.29	—	—	5	0.21

*Note:* Continuous variables are presented as mean and standard deviation (SD). Categorical variables are presented as the number of patients (*N*) and percentage (%).

Abbreviations: CCI, Charlson comorbidity index; DOACs, direct oral anticoagulants; OAC, oral anticoagulant; SIMD, Scottish index of multiple deprivation; VTE, venous thromboembolism.

**FIGURE 2:**
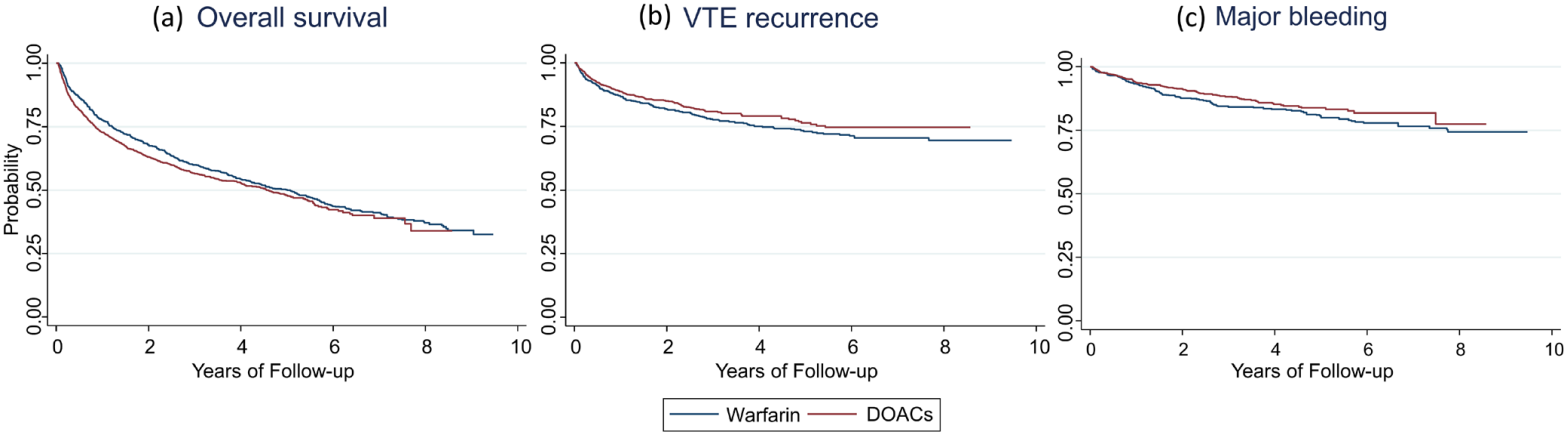
Kaplan–Meier curves of patients on Warfarin or DOACs; (a) Overall survival (b) VTE recurrence (c) Major bleeding. DOACs, direct oral anticoagulants; VTE, venous thromboembolism.

Median OS was longer for patients on warfarin (5.00 years) compared to patients on DOACs (4.61 years). The incidence rate of mortality was 0.14 per 1000 person‐years in the warfarin group and 0.19 per 1000 person‐years in the DOACs group (Figure 2a).

Patients on DOACs had a higher risk of mortality (HR 1.11, 95% CI 0.97–1.26) compared to patients on warfarin, though this was not statistically significant (Table 2). The risk of mortality increased with increasing age except for patients under 60 years. Additionally, patients with active cancer had a higher risk of mortality (HR 1.83, 95% CI 1.49–2.24) compared to patients without active cancer. People with a cancer type associated with a high risk of VTE had a higher risk of mortality (HR 1.63, 95% CI 1.30–2.04) and patients with a cancer type associated with a low risk of VTE (HR 0.67, 95% CI 0.55–0.81) compared to people with other types of cancer. Furthermore, having one or two comorbidities (HR 0.75, 95% CI 0.62–0.90) (reference: no comorbidities) and a moderate to high HAS‐BLED score (HR 0.79, 95% CI 0.66–0.94) (reference: HAS‐BLED—low to moderate risk) were associated with a lower risk of mortality.

**TABLE 2:** Adjusted hazard ratios for DOACs compared to warfarin for all outcomes: mortality, VTE recurrence, and major bleeding.

Covariates	Mortality		VTE recurrence		Major bleeding	
	HR [95% CI]	*p*	sHR [95% CI]	*p*	sHR [95% CI]	*p*
Treatment (DOACs vs. Warfarin)	1.11 [0.97–1.26]	0.140	**0.73 [0.59–0.90]**	**0.003**	**0.68 [0.53–0.88]**	**0.003**
Sex (reference: male)	0.95 [0.82–1.09]	0.456	1.1 [0.87–1.39]	0.413	**0.73 [0.55–0.97]**	**0.032**
**Age (reference: under 60)**						
60–64	0.75 [0.54–1.04]	0.081	0.67 [0.43–1.05]	0.078	1.24 [0.73–2.11]	0.434
65–69	0.87 [0.64–1.19]	0.384	0.63 [0.42–0.96]	0.031	1.03 [0.62–1.72]	0.895
70–74	1.12 [0.82–1.52]	0.471	0.83 [0.55–1.24]	0.367	0.84 [0.50–1.39]	0.488
75–79	1.30 [0.95–1.78]	0.096	**0.67 [0.45–0.99]**	**0.047**	0.89 [0.54–1.46]	0.646
80–84	**1.55 [1.13–2.14]**	**0.007**	0.93 [0.62–1.38]	0.720	1.09 [0.64–1.87]	0.754
Over 85	**2.38 [1.67–3.39]**	**< 0.001**	0.84 [0.51–1.37]	0.474	0.58 [0.28–1.18]	0.132
**SIMD quintile (reference: 1)**						
2	1.03 [0.83–1.28]	0.775	1.01 [0.72–1.43]	0.943	0.87 [0.57–1.35]	0.543
3	1.09 [0.87–1.36]	0.448	0.83 [0.57–1.19]	0.313	0.85 [0.55–1.33]	0.481
4	**1.27 [1.01–1.60]**	**0.039**	0.89 [0.62–1.27]	0.512	0.84 [0.52–1.36]	0.482
5	0.87 [0.69–1.11]	0.261	0.86 [0.59–1.27]	0.448	1.11 [0.72–1.71]	0.630
**Cancer activity (reference: no)**						
Yes	**1.83 [1.49–2.24]**	**< 0.001**	1.15 [0.89–1.49]	0.291	1.05 [0.77–1.42]	0.776
**Cancer type (reference: others)**						
High risk	**1.63 [1.30–2.04]**	**< 0.001**	1.29 [0.99–1.68]	0.060	**0.68 [0.48–0.98]**	**0.036**
Low risk	**0.67 [0.55–0.81]**	**0.001**	0.77 [0.57–1.05]	0.104	1.10 [0.8–1.53]	0.549
**CCI (reference: 0)**						
1–2	**0.75 [0.62–0.90]**	**0.002**	0.98 [0.74–1.28]	0.862	0.74 [0.53–1.04]	0.079
3+	0.97 [0.75–1.27]	0.840	0.94 [0.65–1.37]	0.759	0.89 [0.58–1.35]	0.574
**HAS‐BLED (reference: low to moderate risk)**						
Moderate to high risk	**0.79 [0.66–0.94]**	**0.008**	**1.39 [1.05–1.84]**	**0.023**	**1.89 [1.35–2.65]**	**< 0.001**
**COMPASS‐CAT (reference: low to moderate risk)**						
High risk	1.05 [0.88–1.25]	0.586	0.93 [0.71–1.22]	0.600	0.78 [0.57–1.07]	0.127

*Note:* Bold font indicates statistically significant results (*p*‐value < 0.05).

Abbreviations: CCI, Charlson comorbidity index; CI, confidence interval; DOACs, direct oral anticoagulants; HR, hazard ratio; sHR, sub‐distribution hazard ratio; SIMD, Scottish index of multiple deprivation; VTE, venous thromboembolism.

The probability of VTE recurrence was higher for patients in the warfarin group throughout the follow‐up period. Median time to VTE recurrence could not be observed during the follow‐up period in both treatment groups. The incidence rate of VTE recurrence was 0.06 per 1000 person‐years in the warfarin group and 0.07 per 1000 person‐years in the DOACs group (Figure 2b).

**FIGURE 3:**
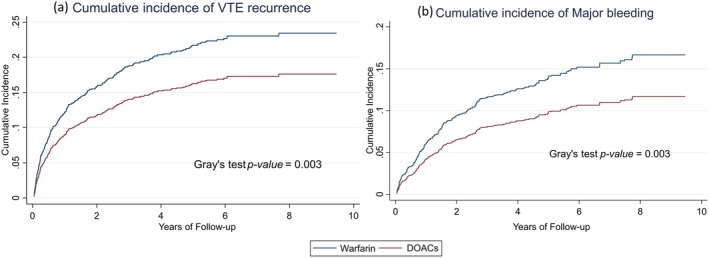
Cumulative incidence of (a) VTE recurrence and (b) Major bleeding with competing risk of death. DOACs, direct oral anticoagulants; VTE, venous thromboembolism.

When death was considered as a competing risk of VTE recurrence, the cumulative incidence of VTE recurrence was higher in the warfarin group throughout the follow‐up period, and the difference was statistically significant (*p*‐value = 0.003) (Figure 3a). The cumulative incidence of VTE recurrence during 12 months of follow‐up was 12.22% in the warfarin group and 9.02% in the DOACs group. Patients on DOACs had a significantly lower risk of VTE recurrence compared to patients on warfarin (HR 0.73, 95% CI 0.59–0.90). A moderate to high HAS‐BLED risk score was associated with a higher risk of VTE recurrence (HR 1.39, 95% CI 1.05–1.84) compared to patients in the low to moderate risk group.

Patients on DOACs had a lower incidence of experiencing a major bleeding event compared to patients on warfarin throughout the follow‐up period (Figure 2c). Median time to major bleeding events could not be observed during the follow‐up period in both treatment groups. Incidence rates for major bleeding events were similar between DOAC and warfarin groups, about 0.04 per 1000 person‐years.

When death was considered as a competing risk of major bleeding, the cumulative incidence of major bleeding was higher in patients on warfarin throughout follow‐up, and the difference between treatment groups was statistically significant (*p*‐value = 0.003) (Figure 3b). The cumulative incidence of major bleeding during 12 months of follow‐up was 6.07% in the warfarin group and 4.19% in the DOACs group. Patients on DOAC showed a significantly lower risk of major bleeding compared to patients on warfarin (HR 0.68, 95% CI 0.53–0.88). In addition to the treatment type, female patients showed a lower risk of major bleeding compared to male patients (HR 0.73, 95% CI 0.55–0.97). A moderate to high HAS‐BLED risk score was associated with a higher risk of major bleeding (HR 1.89, 95% CI 1.35–2.65) compared to patients in the low to moderate risk group. Patients with a cancer type associated with a high risk of VTE had a lower risk of major bleeding (HR 0.68, 95% CI 0.48–0.98) compared to other cancer types.

**FIGURE 4:**
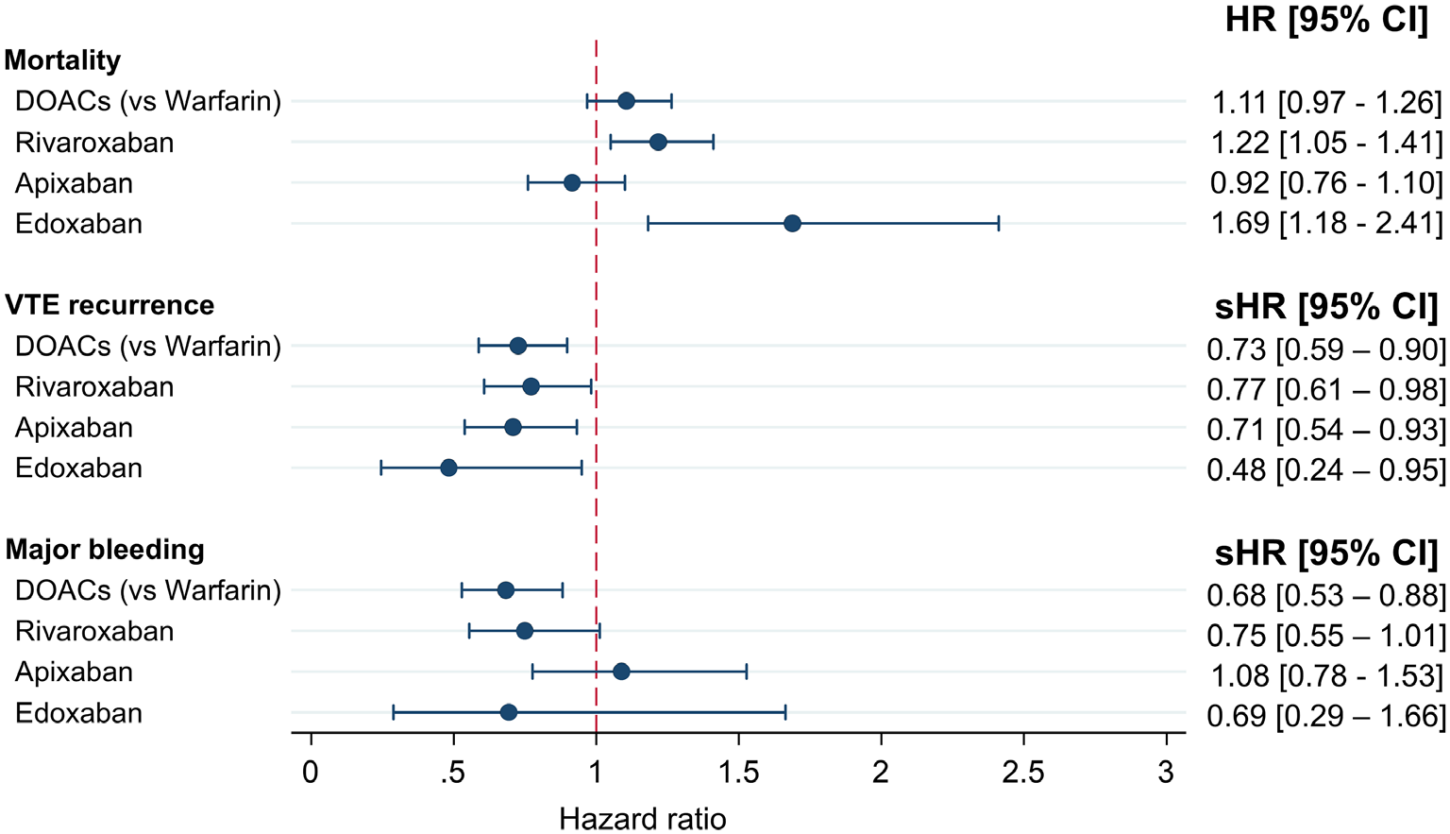
Subgroup analysis on types of DOACs for all outcomes: mortality, VTE recurrence, and major bleeding. CI, confidence interval; DOACs, direct oral anticoagulants; HR, hazard ratio; sHR, sub‐distribution hazard ratio; VTE, venous thromboembolism.

Outcomes were analyzed for patients on individual DOACs, rivaroxaban, apixaban, or edoxaban compared to patients on warfarin (Figure 4). Analysis for the subgroup of patients on dabigatran could not be conducted due to a small sample size. In general, treatment effects were comparable to those of consolidated DOACs. However, there were some differences to highlight.

Patients prescribed rivaroxaban (HR 1.22, 95% CI 1.05–1.41) or edoxaban (HR 1.69, 95% CI 1.18–2.41) had a significantly higher risk of mortality compared to patients on warfarin. In contrast, patients on apixaban compared to patients on warfarin had a lower risk of mortality even though this difference was not statistically significant (HR 0.92, 95% CI 0.76–1.10). The risk of VTE recurrence for patients on individual DOACs compared to patients on warfarin was lowest for patients on edoxaban (HR 0.48, 95% CI 0.24–0.95), followed by apixaban (HR 0.71, 95% CI 0.54–0.93) and rivaroxaban (HR 0.77, 95% CI 0.61–0.98); all individual DOAC groups had a significantly lower risk of VTE recurrence than the warfarin group. The risk of major bleeding was significantly lower for patients on rivaroxaban (HR 0.75, 95% CI 0.55–0.88) compared to warfarin. In contrast, the point estimates for edoxaban (HR 0.69, 95% CI 0.29–1.66) and apixaban (HR 1.08, 95% CI 0.78–1.53) suggest a lower and higher risk, respectively.

## 4 Discussion

This retrospective cohort study utilized administrative linked health datasets from NHS Scotland to assess the comparative effectiveness of DOACs versus warfarin for VTE in cancer patients. Patients on DOACs had a significantly lower risk of VTE recurrence and major bleeding compared to those on warfarin. Looking at individual DOACs, patients on rivaroxaban and edoxaban had a significantly higher risk of mortality in comparison to patients on warfarin, and all types of DOACs were associated with a significantly lower risk of VTE recurrence compared to patients on warfarin.

In previous studies, the inclusion of patients and follow‐up was often limited to specific factors, such as age 65 years and older, few cancer types, and follow‐up periods of less than a year [21, 22, 23]. We were able to include a comprehensive cancer patient population who were prescribed various kinds of OACs for VTE treatment, including those with cancer types associated with a high risk of VTE. Moreover, utilizing long‐term follow‐up data enabled overall survival to be assessed. Even though we attained longer median OS compared to the previous study, we believe that this resulted from the wider scope of patients we included to better reflect clinical practice. To account for potential confounding in this observational study, not only patient‐related factors, but also tumor‐related factors and treatment‐related factors were included in the PS estimation. In addition, we assessed clinically important endpoints such as VTE recurrence, major bleeding, and mortality [30, 31].

In contrast to previous research, our study did not find a significant difference in mortality between patients on warfarin compared to patients on DOACs [21]. However, our results for the risk of VTE recurrence and major bleeding were in line with a review study that showed a beneficial effect of DOACs on VTE recurrence compared to warfarin [22, 23, 46]. Risks of VTE recurrence and major bleeding were significantly lower in the DOACs group.

Tumor‐related factors and treatment‐related factors as well as patient‐related factors appeared to affect mortality, VTE recurrence, and major bleeding. Covariates affecting the outcomes other than treatment type included sex, age, cancer activity, cancer type, CCI, and HAS‐BLED. Female patients were at higher risk of major bleeding compared to male patients. Furthermore, we observed that older patients had a higher risk of mortality except for those under 60. We assumed that the higher mortality of the young age group is due to cancer severity. For some cancer types, such as breast cancer and colorectal cancer, it is known that these are more aggressive and tend to be found at a later stage in younger patients [47, 48]. Patients with active cancer (reference: cancer activity—not active cancer) or cancer types with a high risk of VTE (reference: cancer type—others) had a higher risk of death compared to the respective reference group. Patients with cancers associated with a low risk of VTE (reference: cancer type—others), patients with one or two comorbidities, or a moderate to high HAS‐BLED risk score had a lower risk of death compared to patients with no comorbidity or a low HAS‐BLED risk score, respectively. Conversely, patients with cancer types with a high risk of VTE had a lower risk of major bleeding. Patients in different HAS‐BLED risk score categories showed a unique feature that a moderate to high HAS‐BLED risk score patients had a higher risk of VTE recurrence and major bleeding but a lower risk of death. As the HAS‐BLED risk score is a widely used clinical index, we assume that adequate clinical risk score assessment and following management may have contributed to the reduced risk of mortality. Our study emphasizes that these patient characteristics are significantly associated with the outcomes under investigation. Therefore, it is important to assess patient characteristics thoroughly prior to the decision of treatment type to improve clinical outcomes. To support clinical decision‐making, we suggest that future research further evaluates the association between covariates and outcomes.

In subgroup analyses, individual DOACs showed varying results in terms of effectiveness compared to the warfarin group. All individual DOACs showed a lower risk of VTE recurrence than warfarin regardless of type. This finding was consistent with a randomized trial which found that rivaroxaban was associated with a lower risk of VTE recurrence compared to warfarin [49]. Patients on rivaroxaban and edoxaban had a higher risk of death compared to patients on warfarin, but patients on apixaban had a comparable risk of mortality. This finding suggests that clinicians should consider the potential benefits of individual DOACs.

This study has several limitations. As the data were routinely collected, some clinical variables, such as cancer stage or patient performance status, were not available. In addition, unobserved confounders such as shifting clinical guidelines, physician prescribing preferences, and specific patient‐related factors could not be accounted for. We attempted to mitigate this by using two different risk scores. However, these may not fully capture risks, particularly those with a cancer type associated with a high risk of bleeding, such as acute leukemia or gastrointestinal cancer [50, 51]. We assessed the comparative effectiveness of consolidated DOACs and individual DOACs (rivaroxaban, apixaban and edoxaban), but were unable to include patients who were prescribed dabigatran due to small sample size. Furthermore, to avoid instability in weighting due to underpowered subgroup populations for individual DOACs, the same IPTW weights were applied throughout the study. While this approach ensures methodological consistency, it does not ensure covariate balance within each subgroup and may cause residual imbalance. To mitigate the potential bias from early treatment changes or adverse events, we excluded patients who either switched OAC therapy or experienced study outcomes within the first 14 days of follow‐up. However, the possibility of residual confounding cannot be fully eliminated. Therefore, future research should explore the effects of long‐term treatment continuation and adherence.

## 5 Conclusion

Our study provides insight into the effectiveness of DOACs compared to warfarin for VTE in cancer patients using administrative data from NHS Scotland, reflecting real‐world clinical practice. Patients on DOACs had a lower risk of VTE recurrence and major bleeding compared to patients on warfarin, but our analyses did not show significant differences in the risk of mortality. In subgroup analyses, patients on apixaban showed a comparable risk of death with the warfarin group, while patients on individual DOACs had a lower risk of VTE recurrence compared to patients on warfarin. In light of our findings, we suggest that healthcare professionals consider the potential benefits of individual DOACs when making treatment decisions.

## Author Contributions

**Hye‐In Jung:** conceptualization, formal analysis, funding acquisition, investigation, methodology, software, writing – original draft, writing – review and editing. **Claudia Geue:** conceptualization, project administration, supervision, investigation, methodology, data curation, writing – original draft, writing – review and editing. **Giorgio Ciminata:** formal analysis, investigation, methodology, software. **Eui‐Kyung Lee:** funding acquisition, writing – review and editing.

## Disclosure

Précis: This retrospective cohort study showed that DOACs were associated with lower risks of VTE recurrence and major bleeding in cancer patients treated for venous thromboembolism (VTE) in NHS Scotland from 2009 to 2021. The study suggests that DOACs may offer benefits over warfarin, but treatment decisions should consider individual DOAC profiles.

## Conflicts of Interest

The authors declare no conflicts of interest.

## Supporting information


**Figure S1:** Covariate balance in standardized differences using various adjustment methods: Unadjusted, PS matching (1:2), NN + exact matching (1:2), IPTW. PS, propensity score; NN, nearest neighbor; IPTW, inverse probability of treatment weighting, SIMD, Scottish index of multiple deprivation; CCI, Charlson comorbidity index.

**Table S1:** Indications for oral anticoagulants.
**Table S2:** Types of cancer.

## Data Availability

Data is available only through application to Information Services Division (ISD) via the Public Benefit and Privacy Panel (PBPP). Due to legal restrictions, only researchers listed in the PBPP application can gain access to the data via the National‐Safe‐Haven platform, a secure environment used to maintain the privacy and confidentiality of the personal information held.
