# Supplementary figures and images for: Comparative Effectiveness of Direct Oral Anticoagulants and Warfarin on Venous Thromboembolism in Cancer Patients
Source: Cancer Med. 2025 Sep 9;14(17):e71209. doi: 10.1002/cam4.71209 (PMC12418083; doi:10.1002/cam4.71209)

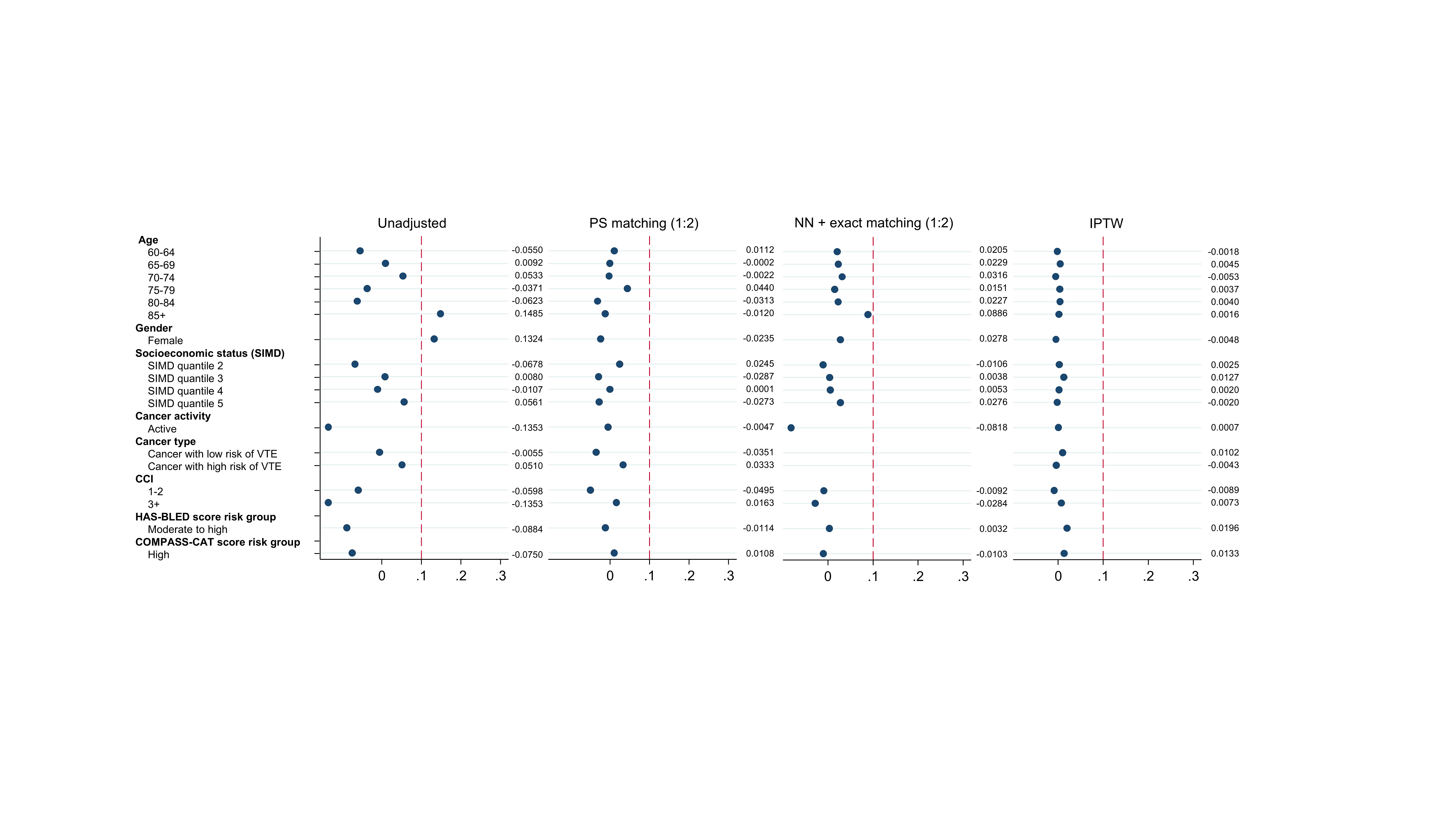

Supplement: Supplementary file 1 — Figure S1: Covariate balance in standardized differences using various adjustment methods: Unadjusted, PS matching (1:2), NN + exact matching (1:2), IPTW. PS, propensity score; NN, nearest neighbor; IPTW, inverse probability of treatment weighting, SIMD, Scottish index of multiple deprivation; CCI, Charlson comorbidity index. [file CAM4-14-e71209-s002.tif]
