# Supplementary material for: Comparative Effectiveness of Direct Oral Anticoagulants and Warfarin on Venous Thromboembolism in Cancer Patients
Source: Cancer Med. 2025 Sep 9;14(17):e71209. doi: 10.1002/cam4.71209 (PMC12418083; doi:10.1002/cam4.71209)
Supplement: Supplementary file 2 — Table S1: Indications for oral anticoagulants. Table S2: Types of cancer. [file CAM4-14-e71209-s001.docx]

Supplementary table 1. Indications for oral anticoagulants

| **Type** | | **Type of code** | **Diagnostic and procedure codes** |
| --- | --- | --- | --- |
| **VTE** | Deep vein thrombosis | ICD-10 | "I636", "I676", "I801", "I802", "I803", "I808", "I809", "I81", "I822", "I823", "I828", "I829" |
|  | Pulmonary embolism | ICD-10 | "I26” |
| **Other**  **indications** | Atrial fibrillation | ICD-10 | "I48" |
|  | Valvular disease | ICD-10 | "I48", "I06", "I07", "I08", "I34", "I35", "I36", "I37", "Q22", "Q23", "Z952", "Z953", "Z954" |
|  | Heart valve replacement | OPCS-4 | "K253", "K273", "K283", "K293" |

Supplementary table 2. Types of cancer

| **Class** | **Cancer type** | **ICD-10 code** |
| --- | --- | --- |
| **High risk cancer** | Stomach cancer | "C16" |
|  | Pancreas cancer | "C25" |
|  | Lung cancer | "C33", "C34" |
|  | Gynaecologic cancer | "C51", "C52", "C53", "C54", "C55", "C56", "C57", "C58" |
|  | Testicular cancer | "C62" |
|  | Kidney cancer | "C64", "C65", "C68" |
|  | Bladder cancer | "C67" |
|  | Brain cancer | "C70", "C71", "C72", "C751", "C752", "C753", |
|  | Lymphoma and myeloproliferative neoplasm | "C81", "C82", "C83", "C84", "C85", "C86", "C88", "C90", "C91", "C92", "C93", "C94", "C95", "C96" |
| **Low risk cancer** | Breast cancer | "C50" |
|  | Prostate cancer | "C61" |
| **Other cancer** |  | "C00", "C01", "C02", "C03", "C04", "C05", "C06", "C07", "C08", "C09", "C10", "C11", "C12", "C13", "C14", "C15", "C17", "C18", "C19", "C20", "C21", "C22", "C23", "C24", "C26", "C30", "C31", "C32", "C37", "C38", "C39", "C40", "C41", "C43", "C44", "C45", "C46", "C47", "C48", "C49", "C60", "C63", "C66", "C69", "C73", "C74", "C76", "C77", "C78", "C79", "C80", "C97" |
